# Sensors, Signals, and Imaging Informatics: Best contributions from 2023

**DOI:** 10.1055/s-0044-1800757

**Published:** 2025-04-08

**Authors:** Leticia Rittner, Christian Baumgartner, Thomas M. Deserno, Section Editors for the IMIA Yearbook Section on Knowledge Representation and Management

**Affiliations:** 1 Medical Imaging Computing Lab (MICLab), School of Electrical and Computer Engineering, University of Campinas, Brazil; 2 Institute of Health Care Engineering with European Testing Center of Medical Devices, Graz University of Technology, Austria; 3 Peter L. Reichertz Institute for Medical Informatics of TU Braunschweig and Hannover Medical School, Braunschweig, Germany

**Keywords:** International Medical Informatics Association Yearbook, Sensor informatics, Signal in-formatics, Imaging informatics, Biomedical informatics, Machine learning, Deep learning, Personalized medicine

## Abstract

**Objectives** : To identify and highlight research papers that represent the advances and trends in the field of sensors, signals, and imaging informatics in 2023.

**Method** : We performed a bibliographic search on Scopus and PubMed databases using Medical Sub-ject Heading (MeSH) terms combined with keywords. Our aim was to build specific queries for sen-sors, signals, and imaging informatics. We disregarded journals that returned less than three papers on the query and then evaluated titles and abstracts of the papers using a 3-point Likert scale, ranging from 1 (do not include) to 3 (should be included). Only the papers with a total score of 8 or more were re-evaluated again, this time considering the full text, and the top 14 papers with the highest scores were then reviewed by external reviewers and editors of the International Medical Informatics Association (IMIA) Yearbook.

**Results** : Among the 643 returned papers published in 2023 in the various areas of sensors, signals, and imaging informatics (SSII), we selected 58 papers with at least 8 Likert points (in total). After a comprehensive evaluation, we identified 14 papers as the best contributions and sent them to eight external reviewers. The full review process resulted in a selection of the four best papers, which were then approved by consensus by the IMIA Yearbook Editorial Board. Although the imaging informatics sub-search returned all of these four papers, one is about sensorless freehand 3D ultrasound recon-struction (representing sensors), and another deals with video-based pulse rate estimation (representing signals).

**Conclusions** : Sensors, signals, and imaging informatics is a dynamic field of intensive research. The four best papers in 2023 represent advanced approaches focusing on DL-based image processing, analysis, and indicate a shift in the research field from sensor technology development to biosignal and image analysis.

## 1. Introduction

Sensors, signals, and imaging informatics (SSII) is a vast field of research that encompasses the acqui-sition, processing, analysis, and interpretation of data. As our annual reviews often show, the number of publications in this area describing approaches based on machine or deep learning-based is growing exponentially. This year, in addition to the growth of publications on DL-based image processing pa-pers, we observe a decrease in publications on sensors. This can be seen as a reduced interest in the development of new sensors technologies but can also be interpreted as a reflection of the replacement of sensor application by sensorless signals and imaging techniques.

Also, the SSII section of the International Medical Informatics Association (IMIA) Yearbook 2023 presents the survey paper entitled “Alzheimer Disease Detection Studies: Perspective on Multi-Modal Data” by Dehghani et al. [ 1 ] This paper explores potential research gaps, challenges, and opportunities related to automated Alzheimer's disease (AD) detection. The authors provided an overview of CAD systems for automated AD detection, focusing on different data types, namely, signals and sensors, medical imaging, and electronic medical records (EMR). As various medical technologies and com-puter-aided diagnosis (CAD), ranging from biosensors and raw signals to medical imaging, have been used to provide information about the state of AD, it may be important for the SSII section to show the challenges and the remaining gaps to identify research opportunities.

## 2. Paper Selection Process

Due to the expanding field of SSII, we had to change the previously used search terms and acronyms [ 2 , 3 ] to obtain a manageable number of returns while including the most relevant publications. We added additional constraints to the previous query and reduced the search terms. As in the previous years, we limited the search to English-language articles and excluded all review articles to streamline the review process and executed the queries on Pubmed and Scopus databases until the end of January 2024. For sensors, signals, and imaging informatics, this search returned 17, 94, and 326 articles from Pubmed as well as 9, 21, and 194 articles from Scopus, respectively. To focus on the most impactful journals, we kept the condition that each journal must have at least three publications in the analyzed subject area to be considered [ 2 3 4 ]. After filtering out duplicates, we obtained 12, 32, and 366 (410 in total) publications.

**Figure 1.**
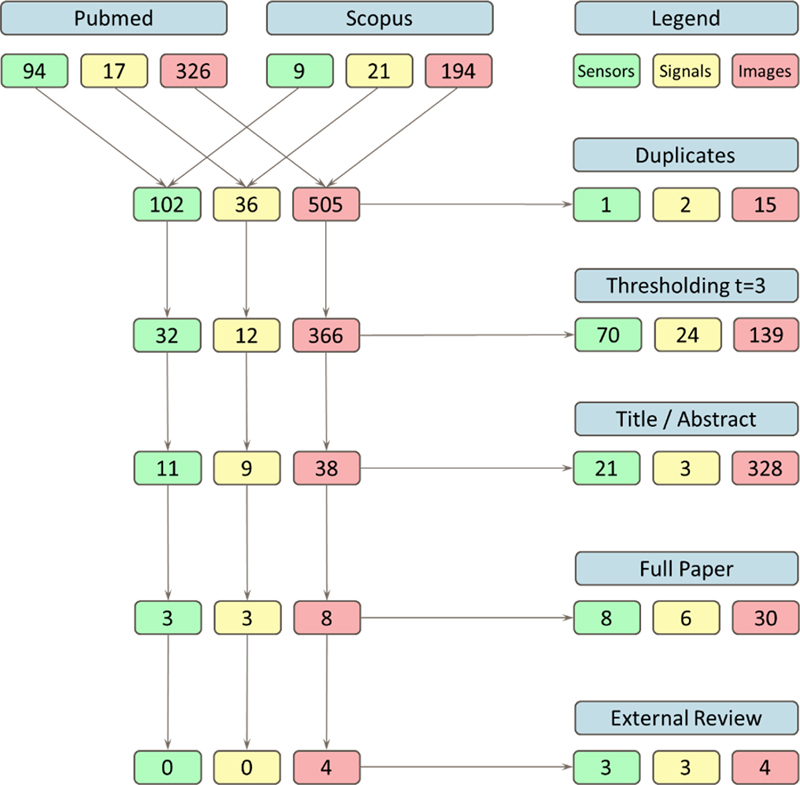
Selection process of the best papers for the 2024 IMIA Yearbook of Medical Informatics for the Sensors, Signals, & Imaging Informatics section.

In the next step, we independently graded titles and abstracts on a three-point Likert scale (1 =  not included, 2 =  maybe included, 3 =  included) and we selected a total of 58 papers with a cumulative score of eight and above ( Figure 1 ). Then, we evaluated the full papers on the same three-point Likert scale and threshold yielding 14 papers for external review.

The total of 14 papers was accessed by eight external reviewers. Based on their ranking, we selected four papers, all of which were from Imaging Informatics ( Table 1 ). However, the clear boundaries between the three fields disappear, as image processing becomes part of modern sensing (as in the selected paper by Luo et al. [ 15 ]) and signal informatics (as in the selected paper by Ouzar et al. [ 17 ]).

**Table 1.** Selection result of best papers for the 2024 IMIA Yearbook of Medical Informatics for the Section Sensors, Signals, and Imaging Informatics. The articles are listed in alphabetical order of the first author's surname. A content summary of these best papers can be found in the appendix of this synopsis.

Section Sensors, Signals, and Imaging Informatics
Li Z, Fan Q, Bilgic B, Wang G, Wu W, Polimeni JR, Miller KL, Huang SY, Tian Q. Diffusion MRI data analysis assisted by deep learning synthesized anatomical images (DeepAnat). Med Image Anal. 2023 May;86:102744. doi: 10.1016/j.media.2023.102744. Chen Y, Lu X, Xie Q. Collaborative networks of transformers and convolutional neural networks are powerful and versatile learners for accurate 3D medical image segmentation. Comput Biol Med. 2023 Sep;164:107228. doi: 10.1016/j.compbiomed.2023.107228. Luo M, Yang X, Wang H, Dou H, Hu X, Huang Y, Ravikumar N, Xu S, Zhang Y, Xiong Y, Xue W, Frangi AF, Ni D, Sun L. RecON: Online learning for sensorless freehand 3D ultrasound reconstruction. Med Image Anal. 2023 Jul;87:102810. Ouzar Y, Djeldjli D, Bousefsaf F, Maaoui C. X-iPPGNet: A novel one stage deep learning architecture based on depthwise separable convolutions for video-based pulse rate estimation. Comput Biol Med. 2023 Mar;154:106592.

## 3. Outlook

Fourteen papers were pre-selected as candidate best papers for 2023, three of them from the field of sensor informatics, three from signal informatics, and eight from imaging informatics.

Of the pre-selected papers from the field of sensor informatics, the first introduces a cuffless blood pressure monitor that does not require periodic calibration. In this study, Wang et al. [ 5 ] showed that the blood pressure monitor generates high-quality photoplethysmographic signals with satisfactory accuracy both at initial calibration and 1-month follow-ups. The paper by Kumaki et al. [ 6 ] was also pre-selected as a best paper candidate, presenting a sheet-type sensor for heartbeat monitoring of sleeping infants and young children, while the third candidate paper by Ortega-Rodríguez et al. [ 7 ] presents a method for reducing the number of electrodes in EEG-based biometric systems for identify-ing individuals by EEG.

As for the field of signal informatics, the pre-selected papers ranged from Covid-19 detection by Celik [ 8 ] based on cough, breath and voice information, emotion recognition by EEG by Qiu et al. [ 9 ], and intelligent antepartum fetal monitoring by Cao et al. [ 10 ]. In the end, all three papers use a DL frame-work to accomplish the proposed task.

Finally, we can categorise the eight papers pre-selected from the field of imaging informatics into three main groups: image segmentation [ 11 12 13 14 ], image reconstruction [ 15 , 16 ] and image analysis [ 17 ]. As in the field of signal informatics, all the aforementioned pre-selected papers on image processing also propose DL-based methods to perform the desired task. The only pre-selected paper in the field of imaging informatics that does not propose a DL-based solution is the one published by Nie et al. that presents a dataset of oral implant image [ 18 ].

## 4. Discussion

While utmost care was placed on the creation of the search queries, the sheer number of publications in SSII made it necessary to limit the scope to achieve a manageable amount or results. This emphasizes the need for continuous refining of the search queries based on emerging technologies, terms, and applications.

Deep learning-based approaches for medical image and biosignal processing dominate the selected papers for this year. Most of these papers deal with medical image processing. Notably, among the top four papers, the first sensorless concepts based on deep learning-based image processing were present-ed. These approaches attempt to replace biosignal information, typically measured by biomedical sen-sors, with image information. Sensorless biosignal processing using ML/DL-based methods thus rep-resents an intriguing and pioneering development in this field.

For the 2025 Yearbook, we plan to rename our section from “Sensors, Signals, and Imaging Informat-ics (SSII)” to “Biosignal and Imaging Informatics (BII)” to better reflect the two SSII subsections—sensors and signals—by the term “biosignal.” The main focus is not on newly developed sensor tech-nology, but on the methods for processing and analyzing the bio-information generated by the sensors, which motivated us to rename the SSII section.

To summarise, the renaming of the section to “Biosignal and Imaging Informatics (BII)” is a strategic move that is in line with the current trends and future direction of the field. This change emphasises the shift from sensor development more to advanced processing and analysis of biosignals and medical images, which ultimately contributes to better patient outcomes and more efficient healthcare delivery.
